# Psychometric evaluation of the Persian version of the Beliefs About Voices Questionnaire: revised in patients with schizophrenia spectrum disorders

**DOI:** 10.1186/s12888-025-07080-z

**Published:** 2025-07-01

**Authors:** Vida Yousefi Asl, Abbas Pourshahbaz, Ali Nazeri Astaneh, Farhad Taremian

**Affiliations:** 1 Psychosis Research Center, Department of Clinical Psychology, Faculty of Behavioral Sciences, University of Social Welfare and Rehabilitation Sciences, Tehran, Iran; 2 Department of Clinical Psychology, Faculty of Behavioral Sciences, University of Social Welfare and Rehabilitation Sciences, Tehran, Iran; 3 Psychosis Research Center, University of Social Welfare and Rehabilitation Sciences, Tehran, Iran; 4 Department of Clinical Psychology, University of Social Welfare and Rehabilitation Sciences, Tehran, 1985713871 Iran

**Keywords:** Omnipotence, Malevolence, Benevolence, Hallucinations, Hearing voices, BAVQ– R, Beliefs about voices

## Abstract

**Background:**

The Beliefs About Voices Questionnaire-Revised (BAVQ-R) is a widely used measure in research and clinical settings designed to assess beliefs about auditory hallucinations and the corresponding response styles. Despite its common usage, the psychometric properties of the BAVQ-R are understudied. This study aimed to contribute to this gap in the literature by examining the psychometric properties of the Persian version of the BAVQ-R in Iran.

**Method:**

A total of 298 patients diagnosed with Schizophrenia Spectrum Disorder (aged 19–50 years; *M* = 37.32, *SD* = 1.14; 85.23% male) were recruited from Razi Hospital in Tehran. Participants completed the BAVQ-R alongside self-report measures (e.g., the Beck Depression Inventory-II [BDI-II] and the Schizophrenia Quality of Life Scale [SQLS]) and underwent interview-based assessments (e.g., the Psychotic Symptom Rating Scales [PSYRATS] and the Calgary Depression Scale for Schizophrenia [CDSS]).

**Results:**

A confirmatory factor analysis supported a 31-item four-factor model comprising two belief subscales (Malevolence/Omnipotence and Benevolence) and two response subscales (Resistance and Engagement), with excellent model fit indices (CFI = 0.99, TLI = 0.99, RMSEA = 0.042). The subscales demonstrated high internal consistency (Cronbach’s *α* and McDonald’s *ω* = 0.91–0.95) and correlated in theoretically consistent ways with external constructs (e.g., measures of hallucinations, delusions, depression, and quality of life).

**Conclusion:**

Findings suggest that the Persian BAVQ-R is a reliable and valid instrument for assessing beliefs about voices and response styles in Iranian patients with Schizophrenia Spectrum Disorder. These findings support its use in both research and clinical settings and suggest avenues for further research to explore its applicability across different populations and settings.

**Clinical trial number:**

Not applicable.

## Background

Auditory hallucinations, commonly referred to as “voices,” are frequent and distressing symptoms found across various mental health disorders [1]. However, these experiences are not limited to clinical settings, as approximately 7% of the general population also reports experiencing these phenomena [2]. The characteristics of these auditory hallucinations — such as their form, content, and impact — tend to be consistent across different groups [3], though individuals’ emotional and behavioral reactions to hearing voices can differ significantly [4]. Higher perceived voice power or omnipotence is linked to greater levels of voice-related distress, as well as increased depression, suicidal ideation, and social distress [5, 6]. Those who interpret their voices as dominant, malevolent, and critical experience a heavier burden and face a higher risk of engaging in aggressive and self-harming behaviors [7, 8].

In this context, Chadwick and Birchwood [4] proposed a cognitive model to explain the maintenance of auditory hallucinations, suggesting that individuals’ emotional and behavioral responses are influenced by both the content and the meaning attributed to the voices. They emphasized that beliefs about the purpose and intent of voices—such as their perceived power (omnipotence) and malevolent or benevolent nature—play a key role in shaping how individuals respond to and cope with these experiences. According to their model, people tend to exhibit two main emotional and behavioral response styles: engagement and resistance. Engagement involves actively interacting with the voices, while resistance involves attempts to argue, shout at, or ignore them. Research shows that these response styles are associated with different levels of distress, with engagement linked to lower depression and anxiety, and resistance correlated with higher levels of these variables [9].

Consequently, given the significance of beliefs about auditory hallucinations, the 30-item Beliefs about Voices Questionnaire (BAVQ; 10) and its revised 35-item version (BAVQ-R; 9) were developed to assess the key dimensions of the voice-hearing experience. The BAVQ-R includes three belief subscales: Omnipotence, which measures beliefs about the perceived power of the voices, and two subscales that measure beliefs about voice intent—Malevolent beliefs (perceptions of negative intent) and Benevolent beliefs (perceptions of positive intent). Additionally, the BAVQ-R features two subscales designed to assess response styles: Engagement and Resistance, with each further divided into emotional and behavioral modes of expression.

Despite its wide usage in research and clinical settings, only one study has examined the factor structure of the 35-item BAVQ-R [11]. This sole study examined the factor structure of the BAVQ-R with 450 participants for belief constructs and 269 participants for response styles, utilizing both confirmatory and exploratory factor analysis. The findings did not support a three-factor belief model. Instead, results indicated that a two-factor model, combining Omnipotence and Malevolence into a single Persecutory belief factor, along with a separate Benevolent belief factor, provided a better fit for the data. Additionally, the study suggested that emotional and behavioral response items should not be separated [11]. While informative, the results from this study need replication due to several limitations. Specifically, Strauss et al. [11] employed the Maximum Likelihood (ML) method as the estimator in their confirmatory factor analyses (CFAs), while the literature suggests that the weighted least squares mean and variance-adjusted (WLSMV) is the most appropriate estimator for ordinal data [12]. Therefore, the factor structure of the BAVQ-R should be re-examined using WLSMV as the estimator. Additionally, participants in this study were drawn from diverse settings (e.g., community mental health teams, the Hearing Voices Network) and different countries, resulting in differences in diagnostic status and educational attainment, indicating that their sample was not homogeneous [11]. Consequently, examining the factor structure of the BAVQ-R among a more homogeneous sample, such as those diagnosed with schizophrenia in which hearing voices is common, would be beneficial for the literature on this measure. Further, Strauss et al. [11] conducted separate factor structure analyses on the belief and response styles subscales of the BAVQ-R, which needs to be addressed in future research. In fact, the five subscales belong to a single BAVQ-R measure, and studies examining its factor structure should include all five subscales in a comprehensive CFA. Finally, there is a dearth of literature on the convergent validity of the BAVQ-R scores which was also neglected in the study by Strauss et al. [11]. Understanding convergent validity is crucial as it ensures the measure correlates well with other established instruments assessing related constructs, thereby affirming its utility and relevance in both clinical and research settings.

## The current study

In an attempt to fill the above-explained gaps in the literature, the present study was designed to test the psychometric properties of the Persian BAVQ-R with a sample of patients diagnosed with schizophrenia spectrum disorder. First, based on prior studies on the BAVQ-R [10, 11], we tested several competing factor structure models to identify the best-fitting structure:

- **Three-factor model**: Malevolence, Omnipotence, and Benevolence.
- **Two-factor model**: Resistance and Engagement.
- **Five-factor model**: Malevolence, Omnipotence, Benevolence, Resistance, and Engagement.
- **Four-factor model**: Malevolence/Omnipotence (combined), Benevolence, Resistance, and Engagement.

Given the scarcity of literature on the factor structure of the BAVQ-R, we did not have a solid hypothesis about the potential fit of any specific factor structure models. However, we acknowledged the potential need for modifications, such as removing items, to achieve an acceptable model fit. Next, we examined the internal consistency of the BAVQ-R and expected to find good to excellent scores for its scores [10, 11]. Finally, we examined the convergent and discriminant validity of the BAVQ-R scores was not considered in the study of Strauss et al. [11]. In this regard, we hypothesized the BAVQ-R Omnipotence, Malevolence, and Resistance subscales to be positively correlated with indices of depression and poor quality of life, while the Engagement subscale was expected to yield significant negative associations with the latter [9].

## Method

### Participants and procedure

This study utilized a cross-sectional design. The sample size was determined in accordance with widely accepted recommendations for conducting factor analysis. According to Muthén and Muthén [13], a sample of around 150 participants is generally sufficient for confirmatory factor analysis (CFA). Moreover, a typical guideline proposes including 5 to 10 participants for each item in a measure, which would suggest a required sample size of 175 to 350 for the 35-item BAVQ-R. Participants in this study were 298 inpatients (aged between 19 and 50 years old; *M* = 37.32, *SD* = 1.14; 85.23% male) who were recruited from Razi Hospital in Tehran and diagnosed with a Schizophrenia Spectrum Disorder based on *DSM-5* criteria through clinical interviews. All participants were receiving psychiatric medication. Concerning demographic characteristics, in terms of marital status, the majority were married (206; 68.2%), with a smaller portion being single (53; 17.6%) and divorced (39; 12.9%). Regarding education level, 23 participants (7.7%) were illiterate, 37 (12.3%) had completed elementary school, 70 (23.4%) had completed middle school, 94 (31.5%) had high school education, 67 (22.4%) had a diploma, 2 (0.7%) had an associate degree, and 5 (1.7%) held a bachelor’s degree.^1^ In terms of employment status, 271 (91.5%) were unemployed, 18 (6.1%) were employed, and 9 (3.0%) were homemakers.

Inclusion criteria required participants to be willing to join the study, while exclusion criteria consisted of any physical or mental incapacity to participate, as judged by the data collectors. Research assistants first explained the study’s objectives to the participants, ensuring confidentiality, and obtained informed consent. Subsequently, interview-based assessments were conducted in a quiet room. The interviews lasted approximately 45 min and were followed by the administration of self-report questionnaires, which took about 40 min to complete. All assessments were conducted under the supervision of a PhD-level researcher. Participants were informed that they could take breaks at any time if they felt fatigued. This study was reviewed and approved by the ethics committee of the University of Social Welfare and Rehabilitation Sciences (Code Number: IR.USWR.REC.1400.292).

## Measures

### Beliefs About Voices Questionnaire-Revised (BAVQ-R; 9)

The BAVQ-R was developed by Chadwick et al. [9] and is a 35-item self-report measure designed to assess the beliefs, emotions, and behaviors associated with auditory verbal hallucinations (hearing voices). It includes three beliefs subscales, including Malevolence, which measures beliefs that the voices are malevolent or harmful; Benevolence, which assesses beliefs that the voices are benevolent or protective; Omnipotence, which gauges beliefs regarding the power and authority of the voices; and two response styles subscales, including Resistance, which captures emotional and behavioral responses aimed at resisting the voices; and Engagement, which reflects emotional and behavioral responses aimed at engaging with the voices. Participants rate their agreement with each item on a Likert scale ranging from 0 (*“disagree”*) to 3 (*“strongly agree”*).

#### Persian BAVQ-R

The translation process of the BAVQ-R from English to Persian involved multiple steps to ensure accuracy and comprehensiveness. First, the original version of the BAVQ-R was translated into Persian by the first author, who is fluent in both English and Persian. This initial translation was then given to an independent translator for back-translation into English. An English-speaking evaluator subsequently compared the back-translated version with the original English version to assess fluency and comprehensiveness. Finally, the translators and the authors reviewed and discussed the translated versions until they reached a consensus on the final Persian version.

### **The** psychotic symptom rating sc**ales (PSYRATS; 14)**

The PSYRATS is a 17-item, five-point scale (0 to 4) clinical assessment tool developed by Haddock et al. [14] to measure the severity and impact of auditory hallucinations and delusions in individuals with psychotic disorders. The PSYRATS consists of two main subscales: the Auditory Hallucinations Subscale (PSYRATS-AH; 11 items) and the Delusions Subscale (PSYRATS-DS; six items). The PSYRATS-AH assesses various dimensions of auditory hallucinations, including frequency, duration, location, loudness, beliefs about the origin of the voices, the amount and degree of negative content, distress, and the disruption to life caused by the voices. The PSYRATS-DS evaluates dimensions of delusional experiences such as preoccupation with the delusions, distress, conviction, disruption to life, and the amount of effort expended to challenge or neutralize the delusional beliefs. The internal consistency indices scores for the PSYRATS scores are shown in Table 3.

### The calgary depression scale for schizophrenia (CDSS; 15)

The CDSS is a 9-item clinician-rated scale developed by Addington et al. [15] to assess the level of depression in individuals with schizophrenia or those at clinical high risk for psychosis. It is specifically developed to distinguish depressive symptoms from negative, positive, and extrapyramidal symptoms associated with schizophrenia. The CDSS focuses on symptoms of depression such as depressive mood, hopelessness, self-deprecation, guilt, insomnia, early wakening, and suicidal ideation. Each item is rated on a scale ranging from 0 (*“absent”*) to 3 (*“severe”*), with higher scores indicating more severe depressive symptoms. The total score provides a measure of the severity of depression. We administered the Persian version of the CDSS which demonstrated acceptable psychometric properties in an Iranian sample [16]. The internal consistency indices scores for the CDSS score are shown in Table 3.

### The Beck depression inventory-II (BDI-II; 17)

The BDI-II is a widely used 21-item self-report measure developed by Beck [17] to assess the severity of depressive symptoms in adolescents and adults. Each item describes a specific symptom or attitude related to depression and is scored on a scale from 0 to 3, with higher scores indicating greater severity of symptoms. The total score is calculated by summing the individual item scores, providing an overall measure of depression severity. We utilized the Persian version of the BDI-II which showed acceptable psychometric properties in an Iranian sample [18]. The internal consistency indices scores for the BDI-II score are shown in Table 3.

### Schizophrenia quality of life scale (SQLS; 19)

The SQLS is a self-report measure specifically designed by Wilkinson et al. [19] to assess the quality of life in individuals with schizophrenia. It consists of 30 items rated on a Likert scale ranging from 0 (*“never”*) to 4 (*“always”*) and includes three subscales: Psychosocial, Motivation and Energy, and Symptoms and Side Effects. The Psychosocial subscale assesses social and psychological aspects of quality of life, including relationships, social activities, and emotional well-being. The Motivation and Energy subscale evaluates the individual’s motivation, energy levels, and ability to engage in daily activities. The Symptoms and Side Effects subscale captures the impact of schizophrenia symptoms and the side effects of treatment on the individual’s quality of life. Each item is rated on a Likert scale, reflecting the frequency or severity of specific experiences related to these domains. Scores for each domain are calculated by summing the item scores within that domain, with higher scores indicating a greater impact on quality of life. In this study, we used the Persian version of the SQLS which showed acceptable psychometric properties in a prior study with an Iranian sample [20]. The internal consistency indices scores for the SQLS scores are shown in Table 3.

### Data analyses

For the confirmatory factor analyses (CFAs), we employed the *lavaan* package [21] in RStudio version 2023.3.1.446 [22]. The analyses were conducted using the weighted least squares mean and variance-adjusted (WLSMV) estimator which is particularly suited for ordinal data [12]. Model fit was evaluated using established criteria for acceptable fit: Comparative Fit Index (CFI; ≥ 0.90), Tucker Lewis Index (TLI; ≥ 0.90), and Root Mean Square Error of Approximation (RMSEA; < 0.08) [23, 24]. Based on prior research on the BAVQ-R [10, 11], we tested several competing factor structure models to determine the best-fitting solution. These included a three-factor model (Malevolence, Omnipotence, and Benevolence), a two-factor model (Resistance and Engagement), a five-factor model (Malevolence, Omnipotence, Benevolence, Resistance, and Engagement), and a four-factor model (combining Malevolence and Omnipotence into a single factor, alongside Benevolence, Resistance, and Engagement).

Next, we assessed the internal consistency of the BAVQ-R scores using Cronbach’s alpha with values *α* ≥ 0.70 being considered acceptable [25] and McDonald’s omega with *ω* ≥ 0.70 being considered acceptable [26]. To evaluate the validity of the BAVQ-R scores, we computed zero-order correlations between the BAVQ-R subscales and theoretically related external measures, including clinician-rated indices of hallucinations, delusions, and depression, as well as self-reported measures of depressive symptom severity and quality of life. The correlation coefficients were interpreted according to Cohen’s [27] guidelines: ≤ 0.30 indicating a small effect size, 0.30-0.50 a medium effect size, and ≥ 0.50 a strong effect size.

## Results

### Confirmatory factor analyses

**Fig. 1:**
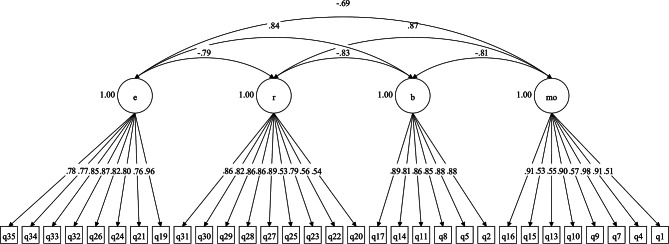
Standardized model parameters for the confirmatory factor analysis: modified four-factor BAVQ-R. Note. *MO* = Malevolence**/**Omnipotence; *B* = Benevolence; *R* = Resistance; *E* = Engagement

As shown in Table 1, the original three-factor model with three belief subscales achieved satisfactory fit based on only two fit indices. Furthermore, Table 2 illustrates that the items related to Malevolence and Benevolence exhibited strong loadings on their respective factors, whereas the items associated with the Omnipotence factor displayed either negative or low loadings. To explore alternative models, we examined a two-factor structure comprising Malevolence/Omnipotence and Benevolence factors. This model also demonstrated adequate fit based on two fit indices. However, a two-factor model incorporating Resistance and Engagement response styles exhibited acceptable fit across all three fit indices. Subsequently, we investigated a five-factor model that combined the three beliefs subscales and the two response style factors. This five-factor model achieved acceptable fit, although Table 2 reveals that the items representing the Omnipotence factor exhibited low or negative loadings on this particular subscale. Next, we explored a four-factor model that incorporated the two beliefs subscales (i.e., Malevolence/Omnipotence and Benevolence) and two response style factors (i.e., Resistance and Engagement), including all 35 items from the BAVQ-R. The results indicated that while this model yielded acceptable fit, items 3, 6, and 18 displayed negative loadings on the Malevolence/Omnipotence factor. Additionally, item 12 had a low loading on this factor. To address this issue, we removed these items from the model and conducted a CFA which resulted in excellent fit (Fig. 1). Consequently, we proceeded with the subsequent analyses using this modified four-factor model.

**Table 1:** Model fit results based on confirmatory factor analyses for models of the BAVQ-R (*n* = 298)

Factor Structure Model	CFI	TLI	RMSEA
Three-Factor Model	0.958	0.951	0.122
Five-Factor Model	0.983	0.982	0.074
Two-Factor Model (MO– B)	0.982	0.978	0.095
Two-Factor Model (R– E)	0.989	0.987	0.070
Four-Factor Model	0.982	0.981	0.077
Modified Four-Factor Model	0.996	0.995	0.042

*Note. RMSEA* = Root Mean Square Error of Approximation; *CFI* = Comparative Fit Index; *TLI* = Tucker-Lewis Index; *BAVQ-R* = Beliefs about Voices Questionnaire– Revised; *MO* = Malevolence**/**Omnipotence; *B* = Benevolence; *R* = Resistance; *E* = Engagement

**Table 2:** Factor loadings of the CFA models for the BAVQ-R (*n* = 298)

Item	Three-Factor			Two-Factor		Two-Factor		Four-Factor				Five-Factor				
	M	B	O	MO	B	*R*	E	MO	B	*R*	E	M	B	O	*R*	E
1	0.860			0.474				0.497				0.505				
2		0.907			0.906				0.878				0.878			
3			0.086					− 0.148						0.082		
4	0.893			0.858				0.890				0.903				
5		0.921			0.920				0.879				0.879			
6			0.079					− 0.173						0.091		
7	0.920			0.896				0.864				0.877				
8		0.865			0.860				0.853				0.853			
9			− 0.297	0.577				0.542						− 0.251		
10	0.553			0.924				0.870				0.884				
11		0.836							0.859				0.859			
12			− 0.085					0.197						− 0.094		
13	0.902			0.575				0.529				0.536				
14		0.765			0.747				0.806				0.806			
15			− 0.266	0.515				0.501						− 0.236		
16	0.860			0.907				0.885				0.898				
17		0.844			0.837				0.890				0.890			
18			0.198	− 0.297				− 0.351						0.178		
19							0.944				0.957					0.958
20						0.566				0.528					0.528	
21							0.794				0.760					0.760
22						0.595				0.560					0.560	
23						0.824				0.791					0.791	
24							0.836				0.800					0.800
25						0.579				0.525					0.525	
26							0.842				0.819					0.818
27						0.863				0.890					0.890	
28						0.871				0.865					0.865	
29						0.861				0.866					0.867	
30						0.812				0.826					0.826	
31						0.816				0.861					0.862	
32											0.865					0.865
33											0.853					0.854
34							0.772				0.769					0.769
35							0.775				0.777					0.777

Note. *BAVQ-R* **=** Beliefs about Voices Questionnaire– Revised; *M* = Malevolence; *B* = Benevolence; *O* = Omnipotence; *MO* = Malevolence**/**Omnipotence; *R* = Resistance; *E* = Engagement

### Descriptive statistics and internal consistency

Descriptive information for the BAVQ-R scores is presented in Table 3. In terms of internal consistency, the *ω* and *α* values ranged from 0.91 to 0.95 for the BAVQ-R scores which are in the excellent range (Table 3).

**Table 3:** Descriptive information for study variables (*n* = 298)

Measures	Mean	SD	Skew	Kurt	ω	α	MIC
BAVQ– R Malevolence/Omnipotence	8.53	7.59	0.51	-1.06	0.91	0.91	0.55
BAVQ– R Benevolence	7.67	6.54	0.15	-1.46	0.94	0.94	0.74
BAVQ– R Resistance	13.47	8.95	0.00	-1.30	0.92	0.92	0.57
BAVQ– R Engagement	6.14	7.76	1.12	− 0.09	0.95	0.95	0.71
PSYRATS Auditory Hallucinations	31.06	6.91	− 0.33	− 0.68	0.78	0.75	0.23
PSYRATS Delusions	20.55	3.21	− 0.68	− 0.17	0.51	0.67	0.29
CDSS	12.74	7.37	0.28	-1.02	0.90	0.89	0.50
BDII	24.95	13.13	0.60	− 0.56	0.94	0.94	0.44
SQLS Total Score	90.01	16.76	− 0.07	− 0.40	0.95	0.95	0.37
SQLS Psychological	47.85	9.71	0.09	− 0.17	0.93	0.93	0.45
SQLS Symptoms and Side Effects	19.28	4.23	0.09	− 0.71	0.89	0.89	0.53
SQLS Motivation and Energy	22.93	4.55	− 0.37	− 0.08	0.82	0.80	0.36

*Note. SD* = standard deviation; *Skew* = skewness; *Kurt* = kurtosis; *α* = Cronbach’s alpha coefficient; *ω =* McDonald’s Omega; *MIC* = mean inter-item correlation; *BAVQ-R* = Beliefs about Voices Questionnaire– Revised; *PSYRATS* = The Psychotic Symptom Rating Scales; *CDSS* = Calgary Depression Scale for Schizophrenia; *BDI– II =* Beck’s Depression Inventory; *SQLS* = Schizophrenia Quality of Life Scale

### Convergent validity

As depicted in Table 4, the BAVQ-R Malevolence/Omnipotence and Resistance subscale scores showed weak to strong significant positive correlations with PSYRATS scores (*r’s* = 0.25 to 0.58), indices of depression symptoms measured by the CDSS and BDI-II (*r’s* = 0.38 to 0.60), and poor quality of life scores (*r’s* = 0.25 to 0.55). Conversely, the BAVQ-R Benevolence and Engagement subscales exhibited moderate significant negative associations with CDSS and BDI-II (*r’s* = − 0.33 to − 0.47) and were weak to strongly correlated with poor quality of life scores (*r’s* = − 0.23 to − 0.51). Additionally, the BAVQ-R Engagement subscale yielded weak to moderate negative correlations with both PSYRATS scores (*r’s* = − 0.24 to − 0.47), whereas the BAVQ-R Benevolence subscale showed a moderate negative correlation only with the PSYRATS Auditory Hallucinations subscale (*r* = −.33).

**Table 4:** Pearson correlation of the BAVQ– R scores with external variables of interests (*n* = 298)

Measures	BAVQ– *R*			
	Malevolence/Omnipotence	Benevolence	Resistance	Engagement
BAVQ– R Malevolence/Omnipotence	1			
BAVQ– R Benevolence	− 0.72**	1		
BAVQ– R Resistance	0.78**	− 0.75**	1	
BAVQ– R Engagement	− 0.63**	0.79**	− 0.72**	1
PSYRATS Auditory Hallucinations	0.58**	− 0.33**	0.55**	− 0.47**
PSYRATS Delusions	0.25**	− 0.10	0.34**	− 0.24**
CDSS	0.38**	− 0.33**	0.53**	− 0.37**
BDI - II	0.44**	− 0.42**	0.60**	− 0.47**
SQLS Total Score	0.42**	− 0.41**	0.55**	− 0.47**
SQLS Psychological	0.44**	− 0.40**	0.53**	− 0.46**
SQLS Symptoms and Side Effects	0.25**	− 0.23**	0.38**	− 0.26**
SQLS Motivation and Energy	0.36**	− 0.42**	0.51**	− 0.51**

*Note. BAVQ-R* = Beliefs about Voices Questionnaire– Revised; *PSYRATS* = The Psychotic Symptom Rating Scales; *CDSS* = Calgary Depression Scale for Schizophrenia; *BDI– II* = Beck’s Depression Inventory; *SQLS =* Schizophrenia Quality of Life Scale; **p <.001

## Discussion

This study aimed to examine the psychometric properties of the Persian version of the BAVQ-R in a homogeneous sample of patients with Schizophrenia Spectrum Disorder in Iran. Our results suggest that the Persian BAVQ-R had a well-supported factor structure that demonstrated good internal consistency coefficients and yielded significant associations with external correlates such as depression and quality of life that are generally consistent with theoretical expectations.

In terms of the factor structure of the BAVQ-R, our results align with the findings of Strauss et al. [11] and cast doubt on the existence of a separate Omnipotence subscale. When examining both three-factor (including Omnipotence, Malevolence, and Benevolence beliefs subscales) and five-factor (including three beliefs subscales and two response style subscales) models, items belonging to the Omnipotence subscale either had low or negative loadings. This partially corroborates the findings of Strauss et al. [11] which did not support the separation of the Omnipotence and Malevolence subscales. Instead, they indicated that a two-factor model, combining Omnipotence and Malevolence items into a single Persecutory belief factor, along with a separate Benevolent belief factor, provided a better fit for the data. Strauss et al. [11] also supported a two-factor model incorporating Resistance and Engagement response styles, though after eliminating two items from the Engagement subscale. Their findings supported a modified BAVQ-R with 29 items from two separate CFAs on beliefs (14 items) and response styles (15 items) subscales. However, it is important to consider that the beliefs and response styles subscales belong to a single BAVQ-R measure, and studies examining its factor structure should include all these subscales in the CFA.

In this vein, we conducted a CFA to examine the fit of a four-factor model, which included all 35 BAVQ-R items loading on two belief subscales (Malevolence/Omnipotence and Benevolence) and two response style subscales (Resistance and Engagement). The results indicated that while this model yielded an acceptable fit, items 3, 6, and 18 displayed negative loadings on the Malevolence/Omnipotence factor, whereas item 12 had a low loading on this factor. Consequently, we removed these four items from the model and conducted a CFA with the remaining 31 items, resulting in an excellent model fit. The poor functioning of items 3, 6, and 18 in our study is consistent with Strauss et al.‘s [11] findings, although item 18 (*“My voice rules my life”*) demonstrated a relatively higher loading in their study. Several factors might explain the poor functioning of this item in our study. The item *“My voice rules my life”* may not align conceptually with the other items in the Persecutory Beliefs subscale, which includes Omnipotence and Malevolence items. While this subscale is intended to measure the perceived power and malevolent intent of the voices, “*My voice rules my life”* could be interpreted more broadly as a statement about the overall impact of the voice, rather than directly reflecting its omnipotence or malevolence. This broader interpretation might lead participants to respond to this item differently than to the more specific items in the subscale, resulting in the observed negative loading. It is also important to note that items 31 and 32, which were removed from the Engagement subscale in the study of Strauss et al. [11], showed high loadings on this factor in our study and were kept in the final factor structure model.

Prior studies have demonstrated that the subscales of the BAVQ-R possess good internal consistency [9–11]. Echoing these studies, our findings indicated that all subscales of the Persian BAVQ-R demonstrated good internal consistency, as measured by *ω* and *α* indices.

To contribute to the scarce literature on the convergent validity of the BAVQ-R scores, we examined correlations of its subscales with external correlates of interest (e.g., indices of hallucinations, delusions, depression, and quality of life). In support of the convergent validity of the BAVQ-R scores, our findings showed that the Malevolence/Omnipotence and Resistance subscales were positively correlated with PSYRATS scores, indicating that higher perceptions of malevolence and omnipotence, as well as greater resistance to voices, are linked to more severe auditory hallucinations. Notably, the BAVQ-R factors showed a stronger correlation with PSYRATS-AH scores compared to PSYRATS-D scores, further supporting the convergent validity of the BAVQ-R scores. Additionally, these subscales were positively associated with depression (CDSS and BDI-II) and poor quality of life, suggesting that negative beliefs about voices and efforts to resist them contribute to increased depressive symptoms and diminished life satisfaction [9]. In contrast, the Benevolence and Engagement subscales demonstrated negative correlations with depression and poor quality of life, highlighting that perceiving voices as benevolent and engaging with them positively is related to lower levels of depressive symptoms and better overall quality of life. These findings suggest that positive interpretations and interactions with voices may help mitigate distress and foster more adaptive coping mechanisms [4]. Furthermore, the Engagement subscale’s negative correlation with PSYRATS scores suggests that actively engaging with voices in a cooperative manner is associated with less severe hallucination symptoms. Similarly, the negative correlation between the Benevolence subscale and the PSYRATS Auditory Hallucinations subscale underscores the beneficial impact of viewing voices as benevolent on reducing the distress and severity of hallucinations [9, 28, 29]. Findings also align with the cognitive model [4], which posits that the interpretation and response to auditory hallucinations play a crucial role in determining the level of distress, depressive symptoms, and quality of life. Negative and resistant attitudes towards voices tend to exacerbate these issues, while positive and engaging attitudes help alleviate them.

### Strengths and limitations

The study’s strengths include the use of a large sample of patients diagnosed with Schizophrenia Spectrum Disorder and the employment of a multimethod approach to data gathering, which integrated both self-report and interview-based measures. This comprehensive approach is crucial for addressing the problem of shared-method variance that arises when relying solely on information from a single informant. Additionally, our study makes a valuable contribution to the limited literature on the factor structure and convergent validity of the BAVQ-R, an area that has been significantly under-researched.

However, several limitations warrant consideration when interpreting the results. First, the reliance on cross-sectional data restricts our ability to infer causal relationships between the variables studied. Second, our sample consisted predominantly of male patients, limiting our findings’ generalizability to female populations. Future research should include larger samples from both genders to enable a more comprehensive examination and to assess the measurement invariance of the BAVQ-R across gender groups. Third, we did not examine the stability of the BAVQ-R factors over time through a test-retest design, which would provide further insight into the instrument’s reliability across different time points. Future research is needed to address this limitation. Fourth, we did not assess variables such as religiosity, social network, and illness duration, which, along with demographic factors, may also influence engagement with voices. Future research should explore the potential role of these variables in understanding voice engagement, as they could offer valuable insights into the complex relationship between voice-hearing experiences and various personal and contextual factors. Finally, our findings are based on a sample of patients with Schizophrenia Spectrum Disorder, limiting the generalizability of these results to patients with other psychiatric diagnoses in which voice-hearing is common. Future studies should aim to include diverse diagnostic groups to broaden the applicability of the findings.

## Conclusion

Overall, our findings indicated that the Persian version of the BAVQ-R, consisting of 31 items, exhibited a robust factor structure and high internal consistency. Additionally, it demonstrated theoretically expected correlations with external correlates of interest. These results support the utility of the BAVQ-R in both research and clinical settings within Iran. Moreover, this study contributes to the existing literature on the BAVQ-R and may encourage further research to enhance the understanding and application of this measure in diverse populations.

### Clinical implications

Our findings have several clinical implications. Notably, the positive correlation between resistant responding to voices and increased levels of depression aligns with prior research [9]. This has important clinical implications, particularly for the development and application of therapeutic interventions [4]. Third-wave cognitive behavior therapies, such as mindfulness-based approaches and Acceptance and Commitment Therapy (ACT), emphasize the potentially counterproductive effects of attempting to resist unpleasant experiences, including auditory hallucinations. These therapies advocate for a non-judgmental acceptance of voice-hearing experiences rather than active resistance. By encouraging patients to accept their voices without judgment, these approaches aim to reduce the distress associated with hallucinations and promote a more adaptive coping style [30]. Our findings, which show that engaging with voices in a positive manner and perceiving them as benevolent are associated with lower levels of depression and better quality of life, further support the efficacy of these therapeutic approaches. By fostering a more accepting and less confrontational relationship with their voices, patients may experience reduced symptoms and improved well-being. In this context, the Brief Coping Strategy Enhancement (CSE; [31, 32]) may also serve as a valuable initial step toward voice acceptance by helping individuals understand and manage their responses before engaging in more complex interventions such as ACT and mindfulness-based strategies. Additionally, Relating Therapy [33], with its aim of developing a more accepting and less confrontational relationship with voices, may also complement these approaches by encouraging individuals to explore more constructive ways of relating to their voices. Therefore, integrating CSE, Relating Therapy, and third-wave approaches could provide a flexible, stepwise framework for supporting individuals experiencing distressing auditory hallucinations.

## Data Availability

The datasets generated during and/or analyzed during the current study are available from the corresponding author on reasonable request.

## Footnotes

1 For participants who were illiterate or had difficulty reading, a trained research assistant read each item aloud in Persian and explained the Likert scale response options to ensure understanding. Participants were encouraged to ask questions if anything was unclear, and their responses were recorded by the assistant. This approach ensured that all participants, regardless of literacy level, could meaningfully engage with the self-report measures.
